# Adjuvant radiotherapy improves cause specific survival in stage II, not stage III mucinous carcinoma of the rectum

**DOI:** 10.1186/s12885-017-3048-4

**Published:** 2017-01-26

**Authors:** Qingguo Li, Yaqi Li, Weixing Dai, Sheng Wang, Ye Xu, Xinxiang Li, Sanjun Cai

**Affiliations:** 1 Department of Colorectal Surgery, Fudan University Shanghai Cancer Center, Shanghai, 200032 China; 2 Department of Oncology, Shanghai Medical College, Fudan University, 270 Dong’an Road, Shanghai, 200032 China

**Keywords:** Rectal cancer, Mucinous adenocarcinoma, Radiotherapy

## Abstract

**Background:**

The effect of adjuvant radiotherapy on the survival outcomes of patients with mucinous rectal cancer remains unclear. This study evaluated the 5-year cause specific survival (CSS) of patients with mucinous rectal cancer after surgery to determine whether adjuvant radiotherapy conferred a survival benefit.

**Methods:**

An analysis of the Surveillance, Epidemiology, and End Results (SEER)-registered database was conducted of patients presenting with mucinous rectal cancer between 2004 and 2011. The primary endpoint was 5-year CSS; univariate and multivariate analyses were performed using Cox proportional hazards regression models.

**Results:**

A total of 574 patients were included for analysis with 248 patients in postoperative radiotherapy group and 326 patients in surgery alone group. Preliminary analysis demonstrated that adjuvant radiotherapy was not associated with CSS (***χ*** ^**2**^ = 0.560, *P* = 0.454). Subgroup analysis indicated that postoperative radiotherapy group had survival advantage in stage II rectal cancer (93.3% vs. 76.6%, ***χ*** ^**2**^ = 4.654, *P* = 0.031), but not in stage III rectal cancer (67.5% vs. 64.7%, ***χ*** ^**2**^ = 0.186, *P* = 0.666). Multivariate analysis demonstrated that postoperative radiotherapy group had a reduced risk of death on survival (HR 0.346; 95%CI 0.129-0.927, *P* = 0.035)

**Conclusion:**

Postoperative radiotherapy is an independent factor for improvement in CSS in patients with stage II rectal mucinous adenocarcinoma, and it should be routinely recommended in these patients. But for stage III patients, considering the losing of CSS advantage and potential radiotherapy toxicity, postoperative radiotherapy should be recommended with great caution.

## Background

Colorectal cancer is the third most commonly diagnosed cancer and the third leading cause of cancer death in men and women combined in the US [1]. Mucinous adenocarcinoma (MC) is a histological subtype of colorectal cancer, representing approximately 5-15% of primary colorectal cancers [2]. In the World Health Organization (WHO) classification, MC is defined as a large amount of extracellular mucin which is produced by secretion from acini and a mucinous layer covering more than 50% of the tumor [3]. MC has a distinct clinicopathological entity with an aberrant molecular background, and it has been reported uniformly associated with younger patient populations, a later stage of presentation, and worse outcomes compared to non-MC [4, 5]. Several studies have reported a poor response of rectal MC to neoadjuvant chemoradiotherapy (NCRT) in terms of downstaging and tumor regression grade [2, 6], while others found a similar survival benefit for MC and non-MC [3].

Although NCRT is the current standard of care for patients with locally advanced rectal cancer, a substantial number of locally advanced rectal cancers have not received NCRT due to understage limited by preoperative evaluation. Data from pooled analyses, as well as from recent smaller studies revealed that the sensitivity of endorectal ultrasound (ERUS) in detecting lymph node metastasis ranges from 50 to 83%, comparable with that of magnetic resonance imaging (MRI) (sensitivity, 45-79%) [7–9]. Adjuvant radiation therapy is recommended for patients with T3, T4, or N+ rectal cancer to decrease the risk of local failure [10], which is reported that about 37% lower in those who had postoperative treatment than those who had surgery alone [11].

Although histopathological type is an important factor predicting tumor response to NCRT [12], the value of adjuvant radiotherapy in MC has not been investigated. Thus, we conducted this study to investigate the prognostic importance of adjuvant radiotherapy in rectal MC.

## Methods

### Patient population

The ideal way to investigate the prognostic factors of a rare disease such as rectal MC is to perform a large population-based study. In this study, we used data from the Surveillance, Epidemiology, and End Results (SEER)-registered database of individuals diagnosed between 2004 and 2011 to explore in detail what aspects of postoperative adjuvant radiotherapy affects MC survival.

The SEER Program of the National Cancer Institute (NCI) is an authoritative source of information on cancer incidence and survival in the United States. SEER currently collects and publishes cancer incidence and survival data from population-based cancer registries covering 28 percent of the US population [13, 14]. The National Cancer Institute’s SEER*Stat software (Version 8.1.5) was used to access the database. The inclusion criteria were as following :(1) patients were diagnosed from 2004 to 2011; (2) the site code represented Rectum (C20.9) according to *Third Edition of International Classification of Diseases for Oncology* (ICD-O-3); (3) histology code denoted MC (8480/3); (4) patients were with no distant metastasis(M0); (5) patients had undergone primary tumor resection; (6) patients received radiotherapy after surgery or surgery alone; (7) patients were at stage II and III; (8) age of patients was limited to above 18 years old; (9) information on cancer-specific survival (CSS) and survival months was available.

### Statistical analyses

Patients’ demographic and clinicopathological variables, including age, sex, race, tumor grade, tumor size, T or N stage, tumor metastatic status, treatment type, reginal lymph node retrieval, et al., were retrieved from the SEER database. The primary endpoint in this study was rectal cancer CSS, defined as the period from diagnosis to death due to rectal cancer. Data of patients who died from other causes or who were alive on the date of their last follow-up was censored.

A comparison of the categorical variables between patients with or without postoperative radiotherapy was conducted using Pearson’s *χ* ^2^ test. The Kaplan-Meier method was used to calculate the actual survival rate and to plot survival curves, followed by the log-rank test for clinical and histological variables. The Cox proportional hazard regression model was used to identify the variables that could independently influence survival in MC. hazard ratios (HRs) and 95% confidence interval (CI) were calculated, with an HR of <1.0 indicating survival benefit. All statistical analyses were performed using SPSS ver.19.0 (SPSS Inc., Chicago, IL), and a value of *P* <0.05 indicated statistical significance.

## Results

### SEER database patient characteristics

**Fig. 1:**
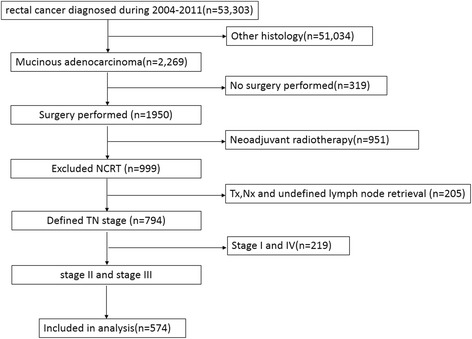
The flow chart of the study

In our 8-year study period, a total of 574 eligible MC patients were enrolled in the current study with a majority of patients being White in race. Figure 1 depicts the flow chart of the study. There were 248 patients in adjuvant radiotherapy group and 326 patients in surgery alone group. The median follow up time was 36 months (0-95 months). The median age at diagnosis was 67 years (range, 25-95 years). Patients with postoperative radiotherapy had a higher rate of young patients, a higher proportion of stage III disease, and relative lower ratio of patients with tumor size less than 5 cm, which reached the level of significance (*P* <0.05). Patient demographics and baseline characteristics are listed in Table 1.

**Table 1:** Characteristics of patients included in the study and comparisons between with and without adjuvant radiotherapy subgroups

		Adjuvant Radiotherapy		*P* value
	Total	No	Yes	
Characteristic	(*n* = 574)	(*n* = 326) (%)	(*n* = 248) (%)	
Sex				0.409
Male	336	186(57.1)	150(60.5)	
Female	238	140(42.9)	98(39.5)	
Age				<0.001
≤60	185	79(24.2)	106(42.7)	
>60	389	247(75.8)	142(57.3)	
Race				0.164
White	492	285(87.7)	207(83.5)	
Black	46	20(6.2)	26(10.5)	
Other	36	21(6.2)	15(6.0)	
Pathological grading				0.378
High/Moderate	395	232(71.2)	163(65.7)	
Poor/Anaplastic	135	71(21.8)	64(25.8)	
Unknown	44	23(7.1)	21(8.5)	
Tumor size(cm)				0.043
<5	253	135(41.4)	118(47.6)	
≥5	277	171(52.5)	106(42.7)	
Unknown	44	20(6.1)	24(9.7)	
LNs retrieval				0.107
<12	185	114(35.0)	71(28.6)	
≥12	389	212(65.0)	177(71.4)	
TNM stage		18(1.6)	5(1.0)	
II	239	163(50.0)	76(30.6)	<0.001
III	335	163(50.0)	172(69.4)	

### Survival impact of postoperative radiotherapy in SEER database

**Fig. 2:**
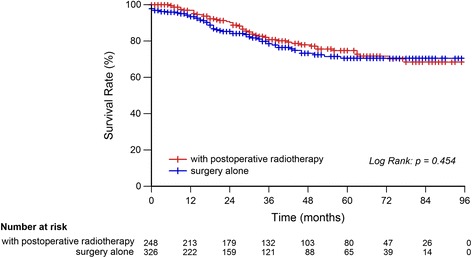
Survival analysis of rectal MC with postoperative radiotherapy or surgery alone. The 5-year CSSs of patients in postoperative radiotherapy group and surgery alone group were 74.8 and 70.5%, respectively, of which the difference was not statistically significant (***χ*** ^**2**^ = 0.560, *P* = 0.454)

**Fig. 3:**
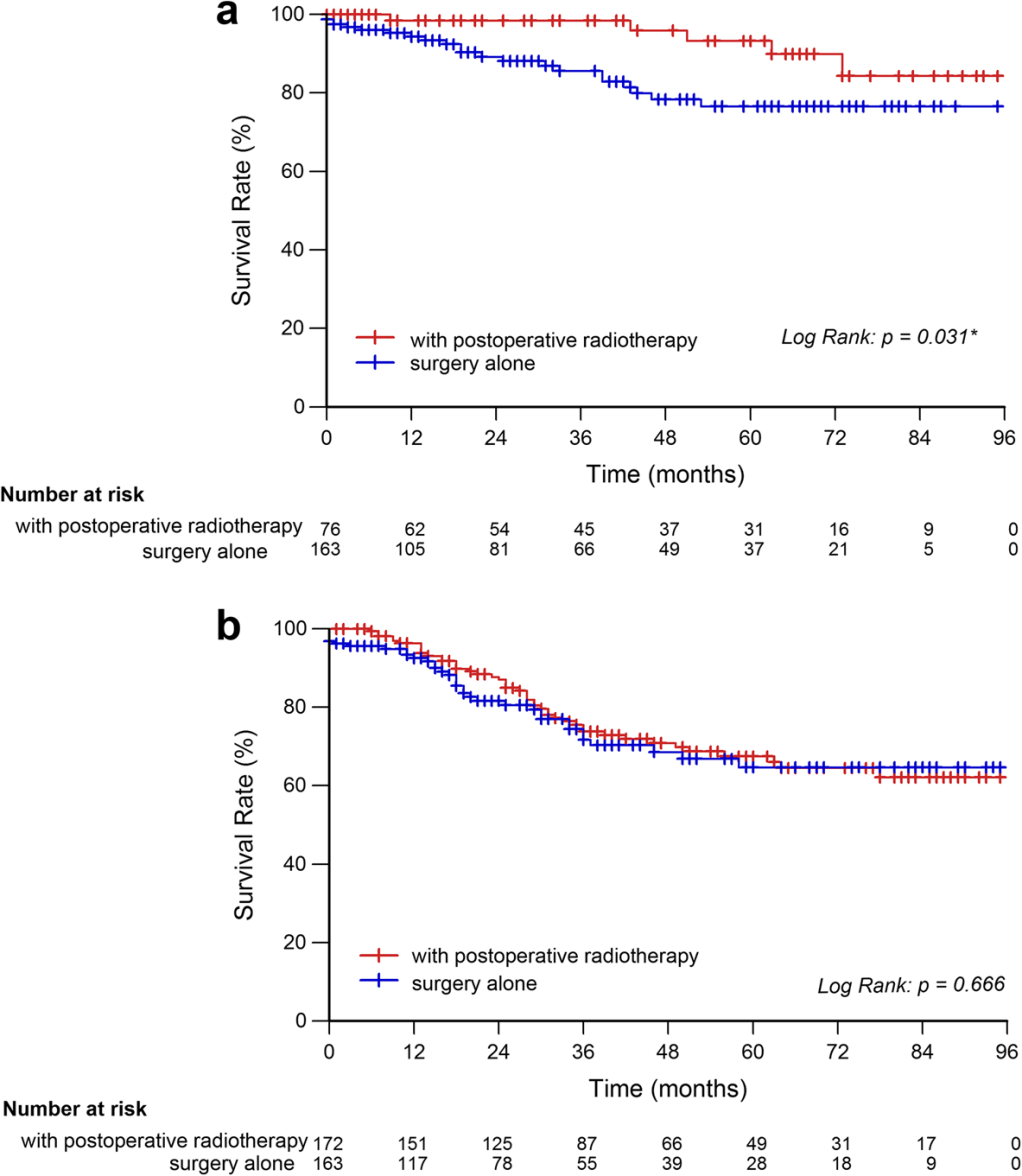
Subgroup analysis the effect of postoperative radiotherapy on rectal MC. The 5-year CSSs of patients in postoperative radiotherapy group and surgery alone group in: (**a**) stage II rectal MC, 93.3% vs. 76.6%, ***χ*** ^**2**^ = 4.654, *P* = 0.031, (**b**) stage III rectal MC, 67.5% vs. 64.7%, ***χ*** ^**2**^ = 0.186, *P* = 0.666

One hundred and eight patients died of rectal cancer at last follow up. The 5-year CSSs of patients in postoperative radiotherapy group and surgery alone group were 74.8 and 70.5%, respectively, of which the difference was not statistically significant (***χ*** ^**2**^ = 0.560, *P* = 0.454) (Fig. 2). Subgroup analysis indicated that postoperative radiotherapy had survival advantage in stage II rectal MC (93.3% vs. 76.6%, ***χ*** ^**2**^ = 4.654, *P* = 0.031), but not in stage III rectal MC (67.5% vs. 64.7%, ***χ*** ^**2**^ = 0.186, *P* = 0.666) (Fig. 3a and b).

The adjuvant radiotherapy (*P* = 0.039), tumor size (*P* = 0.009), and T stage (*P* = 0.040) were significant risk factors for poor survival according to univariate analysis (Table 2). A reduced model was used in the multivariate Cox analysis, which means only variables that were significantly correlated with prognosis in univariate Cox proportion HR analysis were included in the next step. Multivariate analysis demonstrated that tumor size, T stage and adjuvant radiotherapy were independent predictors of CSS and postoperative radiotherapy were found to have a reduced risk of death on survival (HR 0.346; 95% CI 0.129-0.927; surgery alone as reference) (Table 2).

**Table 2:** Univariate and multivariate survival analyses on radiation sequence and cancer specific survival for patients with stage II mucinous rectal cancer

Variable		Univariate analysis		Multivariate analysis	
		HR (95% CI)	*P*	HR (95% CI)	*P*
Sex			0.414		NI
	Male	Reference			
	Female	1.370 (0.643-2.917)			
Age			0.574		NI
	≤60	Reference			
	>60	1.267(0.554-2.898)			
Race			0.942		NI
	White	Reference			
	Black	0.806(0.190-3.413)	0.770		
	Others^*^	0.817(0.110–-6.052)	0.843		
Grade			0.610		NI
	High/Moderate	Reference			
	Poor/Anaplastic	1.096(0.377-3.181)	0.867		
	Unknown	0.374(0.050-2.773)	0.843		
Tumor size(cm)			0.009		0.014
	<5	Reference		Reference	
	≥5	4.142(1.525-11.250)	0.005	3.896(1.428–10.627)	0.008
	Unknown	5.720(1.651-19.817)	0.006	5.251(1.493–18.469)	0.010
LNs retrieval			0.770		NI
	<12	Reference			
	≥12	1.122(0.519-2.424)			
T Stage			0.040		0.028
	T3	Reference		Reference	
	T4	2.487(1.042-5.939)		2.751(1.116–6.780)	
Radiation sequence			0.039		0.035
	No radiation	Reference		Reference	
	After surgery	2.786(1.053-7.371)		0.346(0.129–0.927)	

^*^including other (American Indian/AK Native, Asian/Pacific Islander) and unknown

NI: not included in multivariate survival analysis

## Discussion

In the 1990s, a number of clinical trials found significantly improved rates of local recurrence, cancer-related deaths, and overall survival with adjuvant radiotherapy compared to surgery alone [15–17]. Since then, radiotherapy has become the cornerstone of adjuvant therapy for advanced rectal cancer. During the first decade of the 21st century, preoperative radiochemotherapy with 5-FU became the standard perioperative therapy for locally-advanced rectal cancer due to its higher efficacy in reducing local recurrences and improving disease free survival compared with postoperative radiotherapy, but with no significant advantage in overall survival [18–20]. But for limitation in preoperative assessment, a relative number of patients were understaged, and postoperative radiotherapy was an important complementary. Moreover, most clinical trial only included rectal adenocarcinoma, and the therapy strategies for MC referred to adenocarcinoma. It has been clearly demonstrated that MC is a distinct group of tumors which shows different natural history, biological behavior, different oncogenic and molecular pathways which may make them respond differently to chemoradiation compared to adenocarcinoma [21–23]. However, no current guidelines describe MC as a clinical factor that should influence the therapeutic algorithm. In the era of precise medicine and personalized treatment, it is urgent to know the prognosis value of postoperative radiotherapy in rectal MC.

For low incidence rate of MC in rectal cancer, it will be difficult to accumulate an adequate number of patients treated at a single center. Therefore, we used a large, nationwide, population-based cancer registry, the SEER database, to guarantee power effect for determining the postoperative radiotherapy value of the MC subtype of stage II and III rectal cancer. For there has been great advancements advances in modern radiotherapy in the last decade for rectal cancer [3], we limited the diagnosis from 2004 to 2011 to minimize the confusion. Our study demonstrated that only stage II rectal MC benefit from postoperative radiotherapy, and postoperative radiotherapy was an independently prognostic factor in MC rectal cancer. Considering the toxicity of radiotherapy, postoperative radiotherapy should be recommended with caution for stage III rectal MC.

To our knowledge, the present study is the largest and the first study analyzing the prognostic value of postoperative radiotherapy for MC in stage II and stage III rectal cancer patients. Pooled analysis data from different SEER register centers enabled us to systemically know the protective effect of postoperative radiotherapy in stage II rectal MC. Although this is a large population based study, it should be acknowledged that the result should be interpreted in the light of several limitations. First, there were no information about radiotherapy dosage and additional adjuvant chemotherapy, which may cause confusion. Second, we only selected patients who were pathologically diagnosed as stage II and III rectal cancer and with at least 1 lymph node retrieval to speculate that they had received radical resection. The quality of surgery cannot be assessed in this retrospective study. Third, patients with postoperative radiotherapy has a higher rate of young patients probably with better health and less comorbidities, and therefore, with a better prognosis, which may cause bias to analysis. Fourth, the SEER data lacks the information of local control and relapse free survival, which were the main benefits for radiotherapy in rectal cancer. However, there was defined CSS benefit in stage II rectal MCs who received postoperative radiotherapy, which was sufficient to support the advantage of postoperative radiotherapy in such subgroup of patients. For stage III rectal MC, the possible of local control cannot be converted to CSS advantage.

## Conclusion

According to the findings in our study, we suggest that postoperative radiotherapy should be routinely applied to patients with stage II rectal MC for significantly improved CSS. For stage III patients, considering the losing of CSS advantage and potential radiotherapy toxicity, radiotherapy should be recommended with great caution.

## Abbreviations

CI: Confidence interval
CSS: Cancer-specific survival
ERUC: Endorectal ultrasound
HR: Hazard ratio
MC: Mucinous adenocarcinoma
MRI: Magnetic resonance imaging
NCI: National cancer institute
NCRT: Neoadjuvant chemoradiothrapy
SEER: Surveillance, epidemiology and end results
WHO: World health organization
